# The impact of different combinations of body mass index and depressive status on the incidence of sarcopenia in middle-aged and older adults ≥45 years: A retrospective cohort study based on CHARLS follow-up data from 2011 to 2015

**DOI:** 10.1097/MD.0000000000048695

**Published:** 2026-05-08

**Authors:** Jinmei Lu, Kailei Li, Sumin Wu, Haiming Feng, Zhouzhou Dong, Zaixing Zheng

**Affiliations:** a Department of Critical Care Medicine, Ningbo Medical Center Lihuili Hospital, Ningbo, Zhejiang, China; b Department of Cardiology, Ningbo NO.2 Hospital, Wenzhou Medical University, Ningbo, Zhejiang, China.

**Keywords:** body mass index, China Health and Retirement Longitudinal Study, depression, risk factors, sarcopenia

## Abstract

Sarcopenia is a major public health concern among middle-aged and older adults. Although abnormal body mass index (BMI) and depression are recognized as independent risk factors for sarcopenia, longitudinal evidence regarding their combined impact remains limited. This retrospective cohort study utilized data from the China Health and Retirement Longitudinal Study, including the 2011 baseline survey and 2013 and 2015 follow-up surveys. Participants aged ≥ 45 years without baseline sarcopenia were categorized by BMI (overweight: ≥25 kg/m^2^; non-overweight: <25 kg/m^2^) and depressive status (Center for Epidemiologic Studies Depression Scale-10 ≥ 10). Cox proportional hazards regression model was used to examine associations with incident sarcopenia (Asian Working Group for Sarcopenia 2019 criteria). Among 7845 participants aged ≥ 45 years without baseline sarcopenia, 1294 cases of sarcopenia occurred during a median 4-year follow-up. Participants were classified into 4 groups based on baseline BMI and depressive status: non-overweight without depression (41.0%), non-overweight with depression (21.0%), overweight without depression (26.3%), and overweight with depression (11.7%). Groups differed significantly in sociodemographic characteristics, lifestyle factors, and chronic disease prevalence (*P* < .001). In the multivariable Cox analysis (Model 2, adjusted for sociodemographic, lifestyle, and chronic disease factors), compared with the overweight without depression group (reference), the non-overweight with depression group had the highest risk of developing sarcopenia (hazard ratio [HR] = 2.645, 95% confidence interval [CI]: 2.221–3.150), followed by the non-overweight without depression group (HR = 2.193, 95% CI: 1.858–2.588) and the overweight with depression group (HR = 1.326, 95% CI: 1.066–1.649). Subgroup analysis showed that this association was particularly significant in individuals aged < 65 years (*P* for interaction = .001); the non-overweight with depression group had a 3.395-fold higher risk than the reference group (95% CI: 2.713–4.250). Non-overweight with depression was the highest-risk combination for incident sarcopenia in adults ≥ 45 years, particularly those aged < 65 years. Combined assessment of body composition and psychological status may facilitate early identification and targeted intervention.

## 1. Introduction

Sarcopenia is a progressive, systemic geriatric syndrome primarily characterized by the loss of skeletal muscle mass, muscle strength, and physical function. It has become a major public health concern that seriously threatens the health of middle-aged and older adults.^[1]^ Globally, the prevalence of sarcopenia among older adults is estimated to range from 10% to 16%, with substantial heterogeneity across regions and populations due to variations in diagnostic criteria, age structure, and lifestyle factors.^[2]^ As population aging accelerates in China, the burden of sarcopenia is becoming increasingly prominent. A meta-analysis reported that the pooled prevalence of sarcopenia among community-dwelling older adults in China is 12.9% in men and 11.2% in women, with prevalence increasing markedly with age.^[3]^ Sarcopenia substantially increases the risk of falls, fractures, disability, multiple comorbidities, and all-cause mortality. The resulting health impairments impose considerable clinical and economic burdens on healthcare systems worldwide as well as on patients’ families.^[4–6]^ Consequently, identifying modifiable risk factors for sarcopenia has become a public health priority in China.

Among the multiple modifiable risk factors for sarcopenia, abnormal body mass index (BMI) has been established as an independent risk factor for its development.^[7,8]^ BMI, calculated as weight (kilograms) divided by height (meters) squared, is the most commonly used low-cost and noninvasive indicator for assessing body composition in both clinical practice and research.^[9]^ Although BMI has limitations in accurately identifying abnormal body fat in older adults, it remains a fundamental tool for epidemiological studies and for the clinical screening of sarcopenia.^[10]^ The relationship between BMI and sarcopenia is complex and not strictly linear. On the one hand, the “obesity paradox” has been widely reported among older adults, whereby individuals who are overweight or obese have a lower risk of developing sarcopenia compared with those who are underweight or of normal weight.^[8,11]^ Several cohort studies in Chinese populations have similarly reported that overweight status and moderately elevated BMI are associated with a lower risk of sarcopenia in older adults.^[7,8]^ In contrast, low BMI is a strong independent risk factor for sarcopenia; a BMI < 21.0 kg/m^2^ may significantly predict the onset of sarcopenia across all stages and the progressive decline of skeletal muscle function.^[12]^ However, excessive obesity can accelerate degenerative changes in skeletal muscles through mechanisms such as chronic inflammation, insulin resistance, increased muscle protein breakdown, and inhibition of protein synthesis.^[13]^

Depression is one of the most common mental disorders among middle-aged and older adults in China. The 12-month prevalence of depressive disorders in community-dwelling middle-aged and older Chinese populations ranges from 3.8% to 4.1%, and more than 70% of affected individuals experience varying degrees of social functioning impairment, which substantially affects their quality of life in later years.^[14]^ The 10-item Center for Epidemiologic Studies Depression Scale (CES-D-10) is a concise and validated screening tool widely used to assess depressive symptoms in older adults in China, demonstrating good sensitivity and specificity, with cutoff scores of 10 or 12.^[15,16]^ A meta-analysis of 33 studies involving 119,421 participants reported a significant bidirectional association between depression and sarcopenia. Individuals with sarcopenia had a 2.40-fold higher risk of depression, whereas individuals with depression had a 90% higher risk of developing sarcopenia compared with the general population.^[17]^ Depression may accelerate muscle loss through multiple pathways, including reduced physical activity,^[18]^ insufficient nutrient intake,^[19]^ and activation of the hypothalamic–pituitary–adrenal axis leading to elevated cortisol levels.^[20]^ Conversely, sarcopenia may increase the risk of depression through physical disability, reduced social participation, and chronic inflammation.^[21,22]^

Abnormal BMI and depression often coexist among middle-aged and older adults, and their combined health effects have attracted increasing attention. Individuals with depression often have a lower BMI owing to reduced appetite and insufficient nutrient intake,^[23]^ whereas individuals with obesity tend to have a higher prevalence of depression, which has been associated with presarcopenia.^[24]^ A systematic review suggested that the combined effects of obesity and depression have substantial predictive value for adverse health outcomes; however, the existing evidence remains heterogeneous and limited, particularly in the Chinese population.^[25]^ Moreover, most previous studies have focused primarily on the independent associations between BMI, depression, and sarcopenia. Large-scale longitudinal studies from China examining the combined effects of BMI and depression on the incidence of sarcopenia remain scarce. In particular, few studies have identified which specific BMI–depression combination poses the highest risk for sarcopenia and whether this association is modified by demographic factors such as age.

Therefore, using nationally representative longitudinal data from the China Health and Retirement Longitudinal Study (CHARLS), this study examined the effects of different combinations of BMI and depressive status on the incidence of sarcopenia among adults aged ≥ 45 years. In addition, subgroup analyses were conducted to explore potential effect modifiers. This study aimed to address the existing research gap by providing robust longitudinal evidence on the combined effects of BMI and depressive status on sarcopenia risk and to provide a scientific basis for developing early screening strategies and stratified interventions for sarcopenia among middle-aged and older adults in China.

## 2. Materials and methods

### 2.1. Data source

This retrospective cohort study used data from the CHARLS. Data from the 2011 baseline survey and the 2013 and 2015 follow-up surveys were analyzed. Baseline data were used to define exposure variables and covariates, whereas follow-up data were used to identify incident sarcopenia, thereby establishing a temporal sequence to reduce the risk of reverse causality. CHARLS is a nationally representative prospective cohort study conducted by Peking University that covers 28 provinces in China.^[26]^ The study employs a multistage stratified cluster probability sampling method to collect multidimensional data on adults aged ≥ 45 years. The research team obtained anonymized data via the official CHARLS website on August 31, 2025. Only de-identified data were accessed and analyzed. The CHARLS study was approved by the Biomedical Ethics Committee of Peking University. All procedures were conducted in accordance with the Declaration of Helsinki, and all participants provided written informed consent. Given the use of de-identified, publicly available secondary data, additional ethical approval was waived for the present study.

### 2.2. Selection of study participants

Participants were included if they met all of the following criteria: aged ≥ 45 years; had complete baseline data for sarcopenia assessment, including grip strength, gait speed, 5-time chair stand test results, height, and weight, and were classified as non-sarcopenic at baseline; had complete baseline exposure data, including calculable BMI and a fully completed CES-D-10 scale with no missing items for depression assessment; and had complete sarcopenia-related indicators in either the 2013 or 2015 follow-up waves, allowing identification of incident sarcopenia.

Participants were excluded if they met any of the following criteria: missing key baseline information, including sarcopenia-related indicators, height or weight data required for BMI calculation, or incomplete CES-D-10 responses (≥1 missing item); missing outcome data because sarcopenia-related assessments were not completed in both the 2013 and 2015 follow-up waves, which prevented the determination of incident sarcopenia status.

### 2.3. Definition and measurement of variables

#### 2.3.1. Exposure variable: combined grouping of BMI and depressive status

Using baseline data from 2011, 4 exposure groups were created by cross-classifying 2 binary variables: BMI and depressive status. BMI was calculated from objectively measured height and weight. Participants were categorized as overweight (BMI ≥ 25 kg/m^2^) or non-overweight (BMI < 25 kg/m^2^) according to World Health Organization criteria. Depressive status was assessed using the CES-D-10. A total score of ≥ 10 was used to define depression, consistent with established cutoff values.^[15]^ The 4 exposure groups were overweight without depression, overweight with depression, non-overweight without depression, and non-overweight with depression.

#### 2.3.2. Outcome variable: incident sarcopenia or possible sarcopenia

The outcome was defined as the first diagnosis of sarcopenia or possible sarcopenia during the 2013 or 2015 follow-up waves, based on the Asian Working Group for Sarcopenia 2019 (AWGS 2019) criteria.^[1]^ Muscle strength was assessed using grip strength in both hands. Two measurements were obtained per hand, and the average value was used in the analysis. Low muscle strength is defined as < 28 kg for men and < 18 kg for women. Muscle mass was estimated using a validated anthropometric equation for the Chinese population to calculate appendicular skeletal muscle mass (ASM).^[27]^ The equation was: ASM (kg) = 0.193 × weight (kg) + 0.107 × height (cm) − 4.157 × sex (male = 1, female = 2) − 0.037 × age (years) − 2.631. Low muscle mass was defined using the height-adjusted index (ASM/height^2^) with thresholds of < 7.05 kg/m^2^ for males and < 5.63 kg/m^2^ for females.^[28,29]^ Physical performance was evaluated using gait speed (<1.0 m/s, indicating low speed) and the 5-time chair stand test (≥12 s, indicating low performance). Possible sarcopenia was defined as low muscle strength or low physical performance, whereas sarcopenia was defined as low muscle strength or low physical performance.^[1]^ Incident cases were defined as participants who did not meet these criteria at baseline but were newly diagnosed in 2013 (2-year follow-up) or 2015 (4-year follow-up).

#### 2.3.3. Covariates

Based on previous sarcopenia studies using CHARLS data and considering potential confounding pathways, we included 2 categories of covariates measured at baseline.^[5,30]^ The first category included comprised sociodemographic variables: age (<65 vs ≥65 years), sex, marital status (married/cohabiting vs other), residence (urban vs rural), education (junior high school or below vs high school or above), smoking status (never vs former/current), and drinking status (never vs former/current). The second category consisted of self-reported physician-diagnosed chronic diseases (present vs absent), including hypertension, diabetes, dyslipidemia, heart disease, stroke, chronic lung disease, asthma, arthritis, cancer, memory impairment, psychiatric illness, liver disease, kidney disease, and other digestive disorders.^[26]^

### 2.4. Statistical methods

Statistical analyses were conducted using R software (version 4.4.2), with a 2-tailed *P* < .05 considered statistically significant. Descriptive statistics summarized normally distributed continuous variables as mean ± standard deviations (analyzed using 1-way ANOVA). Non-normally distributed continuous variables as medians and interquartile ranges (analyzed using the Kruskal–Wallis H test), and categorical variables as frequencies and percentages (analyzed using Pearson chi-square or Fisher exact test). The association between BMI–depression combinations and incident sarcopenia was evaluated using Cox proportional hazards regression models, with results reported as hazard ratios (HRs) and 95% confidence intervals (95% CIs). The overweight without depression group was used as the reference. Three models were sequentially constructed: the crude model (unadjusted), Model 1 (adjusted for sociodemographic factors), and Model 2 (additionally adjusted for chronic diseases). The proportional hazards assumption was tested using Schoenfeld residuals, and variables that violated the assumption were addressed using stratified Cox models or time-dependent covariates. Subgroup analyses were performed by sex, age, residence, education, lifestyle factors, and chronic disease status, with interaction terms (*P* < .05) used to assess effect heterogeneity.

## 3. Results

### 3.1. Baseline characteristics of the study participants

**Figure 1.**
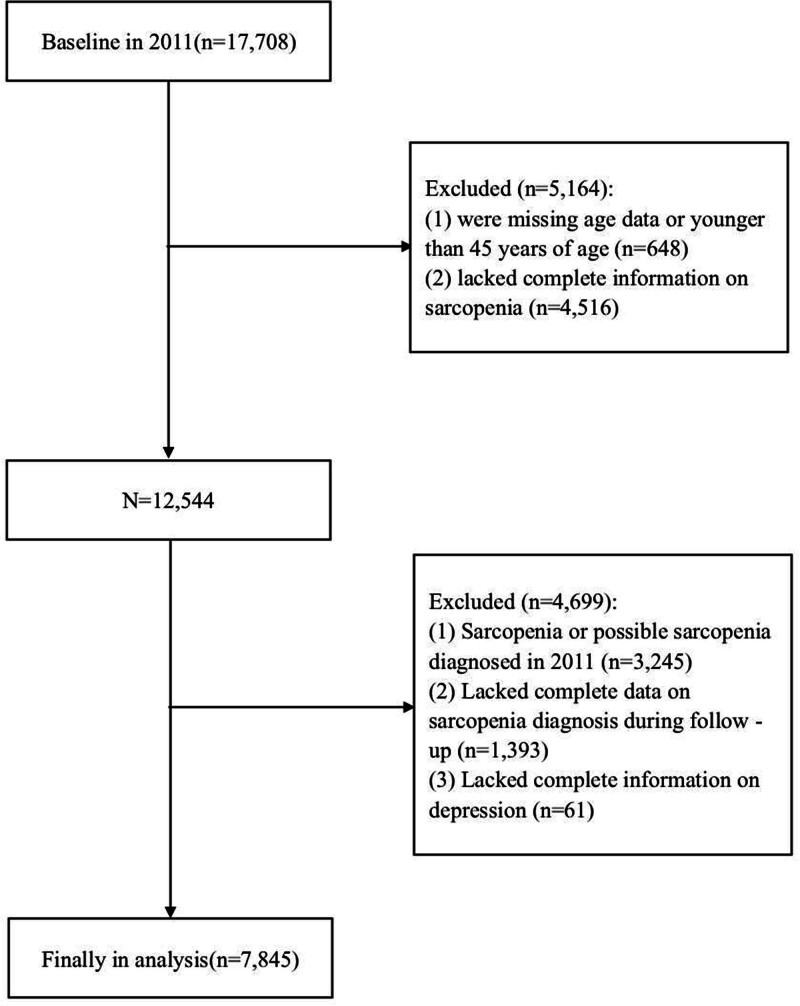
Flowchart of sample selection and exclusion criteria.

Based on the 2011 baseline data from CHARLS, a total of 17,708 participants were initially considered for inclusion. After excluding individuals aged < 45 years, those with missing data, baseline sarcopenia, or incomplete follow-up information, a total of 7845 participants were included in the final analysis (Fig. 1). Participants were categorized into 4 groups according to BMI and depressive status: overweight without depression (26.3%), overweight with depression (11.7%), non-overweight without depression (41.0%), and non-overweight with depression (21.0%). Baseline characteristics differed significantly among the 4 groups (*P* < .001). Notably, the groups with depression had higher proportions of females, rural residents, and individuals with lower educational attainment. In contrast, the overweight without depression group exhibited more favorable health indicators, including higher grip strength and a lower incidence of sarcopenia (9.5% vs 25.5% in the non-overweight with depression group). Detailed results are provided in Table 1.

**Table 1:** Baseline characteristics of the study population in 2011.

	Overall (n = 7845)	Overweight without depression (n = 2067)	Overweight with depression (n = 917)	Non-overweight without depression (n = 3214)	Non-overweight with depression (n = 1647)	*P*-value
Age, >=65years, n (%)	1226 (15.6)	340 (16.4)	173 (18.9)	481 (15.0)	232 (14.1)	.006
Female, n (%)	4006 (51.1)	1157 (56.0)	678 (73.9)	1278 (39.8)	893 (54.2)	<.001
Married, n (%)	7193 (91.7)	1942 (94.0)	807 (88.0)	2996 (93.2)	1448 (87.9)	<.001
Rural living, n (%)	4926 (62.8)	1115 (53.9)	571 (62.3)	2043 (63.6)	1197 (72.7)	<.001
Education, n (%)						<.001
Middle school or below	6891 (87.8)	1751 (84.7)	853 (93.0)	2760 (85.9)	1527 (92.7)	
High school or above	954 (12.2)	316 (15.3)	64 (7.0)	454 (14.1)	120 (7.3)	
Smoking, n (%)	3112 (39.7)	691 (33.4)	220 (24.0)	1544 (48.0)	657 (39.9)	<.001
Drinking, n (%)	3316 (42.3)	794 (38.4)	280 (30.5)	1547 (48.1)	695 (42.2)	<.001
BMI, kg/m^2^	24.0 (21.9,26.4)	27.2 (26.0,28.9)	27.2 (26.0,28.8)	22.5 (21.1,23.7)	22.4 (20.9,23.7)	<.001
Systolic, mm Hg	126.0 (114.0,139.5)	131.0 (119.5145.5)	130.0 (118.0,146.0)	123.0 (112.0,136.0)	121.5 (110.5135.5)	<.001
Diastolic, mm Hg	75.0 (67.5,83.5)	78.5 (71.0,86.5)	78.0 (71.0,87.0)	74.0 (66.5,81.5)	72.5 (65.5,81.0)	<.001
Hypertension, n (%)	1968 (25.1)	719 (34.8)	387 (42.2)	520 (16.2)	342 (20.8)	<.001
Diabetes, n (%)	486 (6.2)	175 (8.5)	104 (11.3)	128 (4.0)	79 (4.8)	<.001
Cancer, n (%)	63 (0.8)	17 (0.8)	13 (1.4)	23 (0.7)	10 (0.6)	.141
Pulmonary diseases, n (%)	639 (8.1)	126 (6.1)	105 (11.5)	229 (7.1)	179 (10.9)	<.001
Heart diseases, n (%)	868 (11.1)	259 (12.5)	182 (19.8)	232 (7.2)	195 (11.8)	<.001
Stroke, n (%)	140 (1.8)	40 (1.9)	37 (4.0)	28 (0.9)	35 (2.1)	<.001
Psychology, n (%)	88 (1.1)	10 (0.5)	23 (2.5)	20 (0.6)	35 (2.1)	<.001
Arthritis, n (%)	2517 (32.1)	614 (29.7)	453 (49.4)	793 (24.7)	657 (39.9)	<.001
Dyslipidemia, n (%)	828 (10.6)	336 (16.3)	172 (18.8)	184 (5.7)	136 (8.3)	<.001
Liver disease, n (%)	261 (3.3)	59 (2.9)	44 (4.8)	79 (2.5)	79 (4.8)	<.001
Kidney disease, n (%)	439 (5.6)	92 (4.5)	70 (7.6)	142 (4.4)	135 (8.2)	<.001
Digestive disease, n (%)	1687 (21.5)	324 (15.7)	257 (28.0)	600 (18.7)	506 (30.7)	<.001
Asthma, n (%)	293 (3.7)	58 (2.8)	64 (7.0)	92 (2.9)	79 (4.8)	<.001
Memory, n (%)	67 (0.9)	18 (0.9)	14 (1.5)	15 (0.5)	20 (1.2)	.004
Depression, n (%)	2564 (32.7)	0 (0.0)	917 (100.0)	0 (0.0)	1647 (100.0)	<.001
CESD-10	6 (3,11)	4 (2,6)	14 (11,18)	4 (2,7)	14 (11,17)	<.001
Low strength, n (%)	0	0	0	0	0	NA
Max grip	34.5 (28.2,41.5)	35.0 (29.0,43.5)	30.0 (25.0,36.0)	36.0 (30.0,43.0)	32.5 (27.0,40.0)	<.001
Low performance, n (%)	3192 (40.7)	903 (43.7)	519 (56.6)	1129 (35.1)	641 (38.9)	<.001
Five-time chair stand test time, s	9.5 (7.8,11.6)	9.6 (7.9,11.7)	10.5 (8.6,13.3)	9.1 (7.4,11.1)	9.8 (7.9,11.8)	<.001
Speed, m/s	0.7 (0.5,0.8)	0.7 (0.5,0.8)	0.6 (0.5,0.7)	0.7 (0.6,0.9)	0.7 (0.5,0.8)	<.001
Low muscle mass, n (%)	789 (10.1)	1 (0.0)	0 (0.0)	457 (14.2)	331 (20.1)	<.001
ASM/Ht2	7.1 (6.2,7.7)	7.5 (6.7,8.4)	7.0 (6.5,8.1)	7.2 (6.0,7.6)	6.3 (5.8,7.4)	<.001
Incident sarcopenia, n (%)	1360 (17.3)	197 (9.5)	146 (15.9)	597 (18.6)	420 (25.5)	<.001

AS = appendicular skeletal muscle mass, BMI = body mass index, CESD-10 = Center for Epidemiological Studies Depression Scale-10, NA = not available; ASM/Ht2, the skeletal muscle mass index (dividing ASM by the square of height).

### 3.2. Association between BMI-depressive status combinations and incident sarcopenia

#### 3.2.1. Survival analysis

**Figure 2.**
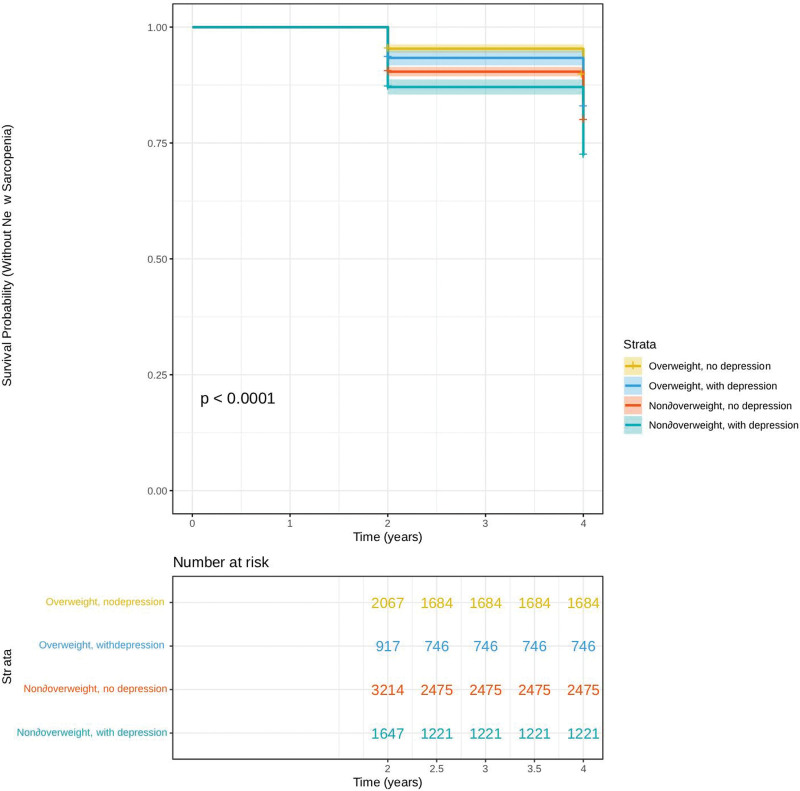
Survival curves of incident sarcopenia across various body mass index and depression subgroups.

During the follow-up period from 2011 to 2015, significant differences were observed in the cumulative incidence of sarcopenia among the 4 BMI–depression groups (log-rank test, *P* < .0001). At the 2-year follow-up, the non-overweight with depression group had the highest incidence of sarcopenia (18.2%), whereas the overweight without depression group had the lowest incidence (4.3%). The overweight with depression (9.7%) and non-overweight without depression (12.5%) groups showed intermediate incidence rates. A similar pattern was observed at the 4-year follow-up. The incidence of sarcopenia reached 25.5% in the non-overweight with depression group, 9.5% in the overweight without depression group, 15.9% in the overweight with depression group, and 18.6% non-overweight without depression group. Overall, these findings suggest that non-overweight individuals with depression have the highest risk of developing sarcopenia. The Kaplan–Meier survival curve illustrating these differences is presented in Figure 2.

#### 3.2.2. Cox proportional hazards regression analysis

Cox proportional hazards regression analyses were performed using the overweight without depression group as the reference category. The results indicated that all other BMI–depression combination groups were associated with a significantly elevated risk of incident sarcopenia across all models. Among these groups, the non-overweight with depression group consistently demonstrated the highest risk. In the crude model, the HRs (95% CIs) were 1.705 (1.377–2.112) for the overweight with depression group, 2.063 (1.756–2.424) for the non-overweight without depression group, and 2.955 (2.495–3.500) for the non-overweight with depression group (all *P* < .05). After adjustment for sociodemographic factors (Model 1), the corresponding HRs (95% CIs) were 1.436 (1.158–1.781), 2.184 (1.855–2.571), and 2.804 (2.361–3.329), respectively. Further adjustment for chronic diseases (Model 2) yielded HRs (95% CIs) of 1.326 (1.066–1.649) for the overweight with depression group, 2.193 (1.858–2.588) for the non-overweight without depression group, and 2.645 (2.221–3.150) for the non-overweight with depression group. All associations remained statistically significant (*P* < .05). Across all models, the non-overweight with depression group consistently demonstrated the highest risk (Table 2).

**Table 2:** Longitudinal analysis of BMI and depression subgroups and incident sarcopenia in the follow-up population.

Variable	n, %	Crude model	Model 1	Model 2
		HR (95%CI)	HR (95% CI)	HR (95% CI)
Overweight without depression	2067 (26.3%)	1 (Ref)	1 (Ref)	1 (Ref)
Overweight with depression	917 (11.7%)	1.705 (1.377–2.112)	1.436 (1.158–1.781)	1.326 (1.066–1.649)
Non-overweight without depression	3214 (41.0%)	2.063 (1.756–2.424)	2.184 (1.855–2.571)	2.193 (1.858–2.588)
Non-overweight with depression	1647 (21.0%)	2.955 (2.495–3.500)	2.804 (2.361–3.329)	2.645 (2.221-3.150)

CI = confidence interval, HR = Hazard ratio; Crude model: no other covariates were adjusted. Model 1: We adjusted for age, gender, marry, rural, Education, smoking, and drinking. Model 2: We further adjusted for Model 1 plus hypertension, diabetes, cancer, pulmonary diseases, heart disease, stroke, psychiatric disorders, arthritis, dyslipidemia, liver disease, kidney disease, digestive disease, asthma, memory disorders.

#### 3.2.3. Subgroup analysis

**Figure 3.**
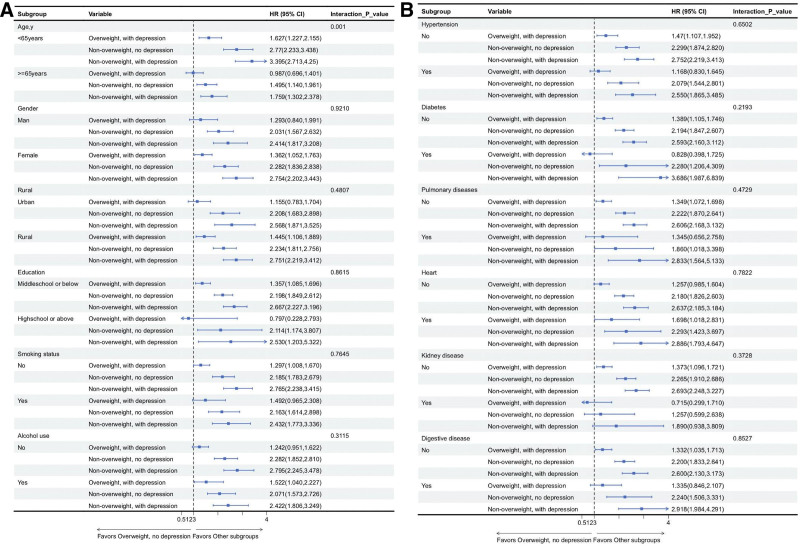
Forest plot of subgroup analyses examining the relationship between various BMI and depression subgroups and incident sarcopenia.HR, hazard ratio; 95% CI, 95% confidence interval; BMI, body mass index.

Subgroup analyses revealed a significant interaction with age (*P* for interaction = .001; Figure 3), whereas no significant interactions were observed for sex, residence, education level, lifestyle factors, or chronic disease status (all *P* for interaction > .05). Among participants aged < 65 years, the risk of incident sarcopenia was significantly higher in all exposure groups compared with the reference group. The HRs with 95% CIs were 1.627 (1.227–2.155) for the overweight with depression group, 2.770 (2.233–3.438) for the non-overweight without depression group, and 3.395 (2.713–4.250) for the non-overweight with depression group. In contrast, among individuals aged ≥ 65 years, the association was not significant for the overweight with depression group (HR = 0.987, 95% CI: 0.696–1.401). However, the risk remained significant for the non-overweight without depression group (HR = 1.495, 95% CI: 1.140–1.961) and the non-overweight with depression group (HR = 1.759, 95% CI: 1.302–2.378), although the effect sizes were smaller than those observed in the younger age group. Across all other subgroup analyses, a consistent pattern was observed: the non-overweight with depression group consistently exhibited the highest risk of incident sarcopenia, and all corresponding HRs (95% CIs) remained statistically significant.

## 4. Discussion

Using data from CHARLS (2011–2015), this study examined the impact of combined BMI and depressive status on the risk of incident sarcopenia among adults aged ≥ 45 years. The results indicated that the non-overweight with depression group had the highest risk of sarcopenia (HR = 2.645, 95% CI: 2.221–3.150), whereas the overweight without depression group had the lowest risk. A significant interaction with age was observed, with consistently higher risks in individuals aged < 65 years, particularly in the non-overweight with depression group (HR < 65 years = 3.395 vs HR ≥ 65 years = 1.759). These findings confirm the association between depression and sarcopenia and further demonstrate that the combined effects of BMI and depressive status, moderated by age, play an important role in sarcopenia risk. Together, these results provide new evidence for stratified prevention of sarcopenia.

Sarcopenia is a progressive skeletal muscle disorder characterized by the loss of muscle mass and function (ICD-10: M62.84).^[31]^ Previous studies have generally examined BMI and depression separately in relation to sarcopenia risk. To our knowledge, this study is the first to evaluate BMI–depression combinations in relation to sarcopenia risk, revealing that the coexistence of non-overweight status and depression substantially increases the risk of sarcopenia. This synergistic association may be explained by several interconnected pathways. Depression may contribute to muscle loss through behavioral and metabolic changes, including poor appetite, inadequate intake of muscle-building nutrients such as protein and vitamin D,^[32]^ and reduced physical activity, which accelerates muscle disuse atrophy.^[23]^ Individuals with non-overweight status typically have lower baseline muscle mass, which may limit their ability to compensate with physiological stressors. Consistent with our findings, Yang et al reported markedly poorer muscle function among individuals with low BMI compared with those with overweight in a CHARLS-based study of adults aged ≥ 60 years.^[33]^ Similarly, Tang et al demonstrated that the risk of sarcopenia increased as BMI decreased, reaching 5.16- and 6.06-fold higher levels in individuals with BMI < 24 kg/m^2^ and < 18.5 kg/m^2^, respectively, which was attributed to metabolic dysregulation along the “muscle–metabolism axis.”^[30]^ These findings align with the elevated risk observed in our non-overweight group with depression. The observed synergy likely arises because insufficient nutrient intake associated with depression and limited muscle reserve associated with non-overweight status jointly impair muscle protein synthesis and promote chronic inflammation (e.g., elevated interleukin -6 and tumor necrosis factor-α), creating a vicious cycle of “muscle loss–metabolic abnormality.”^[32,34]^

Subgroup analyses further identified age as a significant effect modifier (*P* for interaction = .001). The risk associated with BMI–depression combinations, particularly in the non-overweight with depression group, was markedly higher among adults aged < 65 years (HR = 3.395) than in those ≥ 65 years. This aligns with the findings of Zeng et al, who reported a stronger association between depression and sarcopenia in middle-aged (<60 years) than in older adults in analyses of CHARLS data.^[35]^ This pattern suggests a greater susceptibility of younger individuals to depression-induced behavioral changes (e.g., reduced activity and malnutrition) and biological stress (e.g., inflammation), whereas age-related physiological muscle loss in older adults may obscure the independent effect of depression.^[36]^ Furthermore, Zhang et al demonstrated that sarcopenia predicts new-onset multimorbidity, particularly among individuals younger than 60 years (odds ratio = 1.29).^[5]^ Therefore, the elevated risk observed among individuals aged < 65 years with both non-overweight status and depression may represent an early warning signal for future adverse health outcomes, underscoring the importance of targeted preventive strategies.

These findings have important implications for the clinical management of sarcopenia. First, a combined screening approach integrating BMI and depressive status may improve early risk identification. Given the particularly highest risk observed among individuals with non-overweight status and depression (HR = 2.645), a profile often overlooked because of “normal”, individuals with BMI < 25 kg/m^2^ and CES-D-10 ≥ 10 should be prioritized for functional assessments such as grip strength and gait speed to facilitate early risk stratification. Second, interventions should be individualized. For high-risk individuals aged < 65 years, comprehensive strategies should be intensified, including resistance training and adequate protein supplementation^[37,38]^ to improve muscle mass and strength, together with psychological interventions targeting depressive symptoms. For individuals aged ≥ 65 years, clinical management should emphasize ongoing monitoring of muscle function to counter the combined effects of age-related and depression-driven muscle loss. Finally, these results support updating sarcopenia risk assessment frameworks to include depressive status and BMI to enhance predictive accuracy. Concurrently, primary training care should be strengthened to improve the identification of this high-risk profile, thereby enabling early intervention to reduce the incidence of sarcopenia and related adverse outcomes such as falls.

Future research should focus on clarifying the mechanisms underlying the BMI–depression–sarcopenia relationship. The markedly elevated risk observed in the non-overweight with depression group (HR = 2.645) suggests a synergistic interaction, although the underlying pathways remain incompletely understood. Future studies should integrate biomarker detection and functional analyses, including measurements of inflammatory markers and cortisol, to determine whether depression contributes to muscle breakdown through chronic inflammation or stress responses.^[20,34]^ Furthermore, examining the mediating roles of nutrient intake (e.g., protein intake) and physical activity may determine whether non-overweight status increases sarcopenia risk by limiting substrates for muscle synthesis or promoting disuse. Incorporating muscle metabolomics may also reveal whether these factors alter energy metabolism (e.g., mitochondrial function), thereby providing mechanistic insights that may inform the development of targeted preventive interventions.

This study has several limitations. First, the reliance on self-reported data for depressive symptoms (CES-D-10) and chronic diseases may introduce information and recall bias despite the validated use of the CES-D-10 in this population.^[16]^ Second, sarcopenia was diagnosed based on anthropometric equations rather than direct dual x-ray absorptiometry (DXA) measurement for muscle mass assessment; although validated against DXA,^[27]^ this method remains less accurate. Third, unmeasured confounders, such as detailed dietary intake and physical activity intensity levels, may have affected the observed associations. Fourth, the 4-year follow-up period limited the ability to evaluate long-term sarcopenia progression and related adverse outcomes, such as fractures and disability. Finally, the lack of biomarker data (e.g., inflammatory markers and cortisol) prevented further exploration of the underlying biological pathways.

## 5. Conclusions

Using longitudinal data from CHARLS, this study identified the co-occurrence of non-overweight status and depression as the highest-risk profile associated with incident sarcopenia in adults aged ≥ 45 years, with a particularly pronounced association among those aged < 65 years. Incorporating BMI and depressive status into risk assessments is essential for understanding the etiology of sarcopenia and for developing targeted interventions.

## Acknowledgments

The authors thank all members of CHARLS and the participants who contributed to the data. Zhouzhou Dong and Zaixing Zheng contributed equally as co-corresponding authors to this work. Correspondence regarding the methodology and data analysis of this manuscript can also be addressed to Zhouzhou Dong at lhldongzhouzhou@nbu.edu.cn.

## Author contributions

**Conceptualization:** Jinmei Lu, Sumin Wu, Haiming Feng.

**Data curation:** Jinmei Lu.

**Formal analysis:** Kailei Li.

**Methodology:** Jinmei Lu.

**Project administration:** Zhouzhou Dong, Zaixing Zheng.

**Supervision:** Zhouzhou Dong, Zaixing Zheng.

**Validation:** Haiming Feng.

**Writing – original draft:** Jinmei Lu, Kailei Li.

**Writing – review & editing:** Jinmei Lu, Sumin Wu, Zhouzhou Dong, Zaixing Zheng.
